# ADHD Symptoms and Educational Level in Adolescents: The Role of the Family, Teachers, and Peers

**DOI:** 10.1007/s10802-023-01047-y

**Published:** 2023-03-23

**Authors:** Heiko Schmengler, Margot Peeters, Gonneke W. J. M. Stevens, Catharina A. Hartman, Albertine J. Oldehinkel, Wilma A. M. Vollebergh

**Affiliations:** 1 Department of Interdisciplinary Social Science, Utrecht University, Padualaan 14, Utrecht, 3584 CH The Netherlands; 2 Interdisciplinary Center Psychopathology and Emotion Regulation, Department of Psychiatry, University Medical Center of Groningen, University of Groningen, Groningen, 9700 RB The Netherlands

**Keywords:** ADHD, Education, Family functioning, Social support, Teachers, Peers

## Abstract

Few studies have explored the contribution of family and school factors to the association between ADHD symptoms and lower education. Possibly, having more ADHD symptoms contributes to poorer family functioning and less social support, and consequently a lower educational level (i.e., mediation). Moreover, the negative effects of ADHD symptoms on education may be stronger for adolescents with poorer family functioning or less social support (i.e., interaction). Using data of the Dutch TRAILS Study (*N* = 2,229), we evaluated associations between ADHD symptoms around age 11 and educational level around age 14, as well as between ADHD symptoms around age 14 and 16 years and subsequent changes in educational level around age 16 and 19, respectively. We assessed the potential mediating role of family functioning, and social support by teachers and classmates, all measured around ages 11, 14, and 16, while additionally evaluating interactions between ADHD symptoms and these hypothesized mediators. ADHD symptoms were associated with poorer family functioning, less social support by teachers and classmates, and lower education throughout adolescence. No conclusive evidence of mediation was found, because unique associations between family functioning and social support by teachers and classmates and education were largely absent. Furthermore, we found no interactions between ADHD symptoms and family functioning and social support by teachers and classmates. Although social support by teachers and classmates and good family functioning may benefit the wellbeing and mental health of adolescents with high levels of ADHD symptoms, they will not necessarily improve their educational attainment.

**Supplementary Information:**

The online version contains supplementary material available at 10.1007/s10802-023-01047-y.

## Introduction

Symptoms of attention-deficit/hyperactivity disorder (ADHD) are associated with significant impairments in adolescents’ academic functioning, as well as a lower level of education (Schmengler et al., 2021). In selective educational systems like in the Netherlands, this association begins in childhood, when children with high levels of ADHD symptoms are more frequently assigned to lower educational tracks. Selective educational systems are defined by an early selection into different educational tracks while allowing for mobility between tracks post-selection. Subsequent to this initial selection, symptoms of ADHD have been consistently associated with moving to a lower educational track throughout all phases of adolescence and in young adulthood (Schmengler et al., 2021).

While the association between ADHD symptoms and lower education is well-established, little research has focussed on risk or protective factors in the social context, which may contribute to this association (Dvorsky & Langberg, 2016; Dvorsky et al., 2018; Zendarski et al., 2017). This is surprising, considering that it is well known that adolescents with high levels of ADHD symptoms commonly experience problems in their relationships with parents, teachers, and peers (Ewe, 2019; Glatz et al., 2011; McQuade, 2020). Meanwhile, studies have consistently highlighted the importance of these relationships for adolescents’ academic development in general (Lin et al., 2019; Robertson & Reynolds, 2010; Roorda et al., 2017; Tao et al., 2022; Wentzel et al., 2021). It is therefore plausible that poorer social relationships with parents, teachers, and peers might act as mechanisms (mediators) contributing to the association between ADHD symptoms and lower education. Furthermore, adolescents with relatively poor relationships with their family, peers, or teachers and high levels of ADHD symptoms may be at a particularly high risk of poor educational outcomes, which would imply interactions between ADHD symptoms and adolescents’ social context. From a clinical perspective, it is critical to explore these interactions to identify especially vulnerable subgroups, who should be prioritized for interventions.

A better understanding of social relationships in the context of ADHD and educational outcomes may also be informative for the development of new psychosocial interventions, as previous studies have highlighted the benefits of involving both the family and school to achieve positive outcomes (DuPaul et al., 2020). For example, parent-teen behaviour therapy has yielded stronger reductions in ADHD symptom severity, as well as in impairments in organization, time management, and planning in the home setting than treatment as usual (Sibley et al., 2016). A peer-delivered intervention prevented declines in class attendance, organization skills, and academic motivation throughout the school year in adolescents with symptoms of ADHD (Sibley et al., 2020). Lastly, a strong student–teacher relationship was one of the most frequently endorsed facilitators by teachers for the use of behavioural classroom interventions for ADHD (Lawson et al., 2022). In this study, we aimed to evaluate the role of three important family and school factors that could be targets in psychosocial interventions (i.e., family functioning, and social support by classmates and teachers) as mediators in the association between ADHD symptoms and (changes in) adolescent educational track in Dutch adolescents, whilst also taking into account potential interactions between ADHD symptoms and these family and school factors.

### Family and School Factors as Mediators in the Association between ADHD Symptoms and Lower Educational Level

While the evidence for the direct and harmful effects of primary ADHD symptoms on adolescents’ education is strong (Schmengler et al., 2021), ADHD symptoms might also affect educational outcomes indirectly, by causing impairments in aspects of adolescents’ social context important for their academic development, such as relationships with parents, teachers, and peers. Indeed, extensive research has characterized the harmful impact of ADHD symptoms on these relationships, which may lead to poorer family functioning and receiving less social support at school.

In the parental home, adolescents with high levels of ADHD symptoms frequently have difficulties following directions and are less responsive to cues and punishment, rendering parental rule-setting less effective (Glatz et al., 2011). This can lead to perceptions of powerlessness in parents (Glatz et al., 2011), who may react with less responsiveness and emotional support (Glatz et al., 2011; Jones et al., 2015). In families with adolescents with high levels of ADHD symptoms, studies reveal more parental stress, marital conflict, higher rates of divorce, as well as poorer overall family functioning (Moen et al., 2014; Schroeder & Kelley, 2009; Theule et al., 2010; Wiener et al., 2016; Wymbs et al., 2008).

The classroom context, which requires students to sit still and pay attention, can make ADHD symptoms particularly salient. In comparison to their typically developing peers, adolescents with high levels of ADHD symptoms show significantly shorter attentive states during class, more off-task behaviours, and less overall engagement in school (Rogers et al., 2015). Often teachers lack awareness of ADHD, leading them to believe that students’ inattentive and impulsive behaviour is intentional (Wiener & Daniels, 2015), and they may hence resort to frequent criticism and disciplinary penalties, leading to further negative responses from adolescents (Honkasilta et al., 2016). Indeed, studies report more conflictual and less emotionally close relationships between students with ADHD and their teachers (Ewe, 2019).

ADHD symptoms can also cause difficulties in relationships with peers. For example, adolescents with ADHD more often have problems waiting for their turn in give-and-take exchanges, often talk excessively, and more frequently interrupt others (McQuade, 2020). Restlessness and fidgeting may be misinterpreted as disinterest or impatience (McQuade, 2020). As a result of these disruptive behaviours, adolescents with ADHD symptoms tend to report fewer friends, and are more commonly rejected by their classmates (McQuade, 2020; Wiener & Daniels, 2015).

Impairments in the family life and in relationships with teachers and peers caused by ADHD symptoms may adversely affect academic performance, and in this way contribute to the association between ADHD symptoms and lower educational attainment. Past research has highlighted poor social relationships within the family and with teachers and peers as risk factors for academic problems and poor educational outcomes. These studies have mainly focussed on academic development in general, and not specifically in the context of ADHD. For example, it was found that adolescents from poorly functioning families tend to have lower math, logic, and reasoning skills (Lin et al., 2019), lower school engagement and academic self-efficiency (Stubbs & Maynard, 2017), higher risks of school disruption (Sun et al., 2021), lower academic achievement (Blackson, 1995), and ultimately lower educational attainment (Robertson & Reynolds, 2010; Roy et al., 2017). The less effective parenting styles that frequently characterize poorly functioning families may act as a mechanisms connecting family dysfunction and poorer adolescent educational outcomes (Chan & Koo, 2010; Lin et al., 2019; Matejevic et al., 2014; Spera, 2005).

Accordingly, it was found that lack of social support by or poor relationships with teachers predict worse academic outcomes. Meta-analyses found associations of the quality of teacher-student relationships and social support from teachers with academic achievement (Roorda et al., 2017; Tao et al., 2022). Meta-analytic mediation analyses revealed that the association between social support from teachers and academic achievement was partially driven by behavioural (participation in academic activities, e.g., homework completion), emotional (feelings about school/academics, e.g., interest, enjoyment, school belonging and identification), and cognitive engagement (level of psychological investment in academics, e.g., intrinsic motivation, self-efficacy, self-regulation, learning strategies, goals and values) (Tao et al., 2022). Also, social support by and the quality of relationships with peers have been associated with adolescents’ educational outcomes (Ahmed et al., 2010; Fang et al., 2020; Lorijn et al., 2022; Wentzel et al., 2021; Woodward & Fergusson, 2000). Adolescents who do not perceive much support from their peers tend to be less motivated to learn new skills, enjoy studying less, feel less competent and interested in subject-matter knowledge, and are less able to cope with academic difficulties, which in turn was found to predict lower academic achievement (Ahmed et al., 2010; Fang et al., 2020; Patrick et al., 2007).

Regarding children and adolescents with high levels of ADHD symptoms, one study demonstrated that parental marital problems in childhood (which could be indicative of poor family functioning) are associated with lower educational attainment in young adults with a previous ADHD diagnosis (Roy et al., 2017), and one study showed that not having a close bond with teachers is associated with lower academic motivation (Rogers et al., 2015). While these two studies demonstrated associations between family and school factors and education amongst children and adolescents with ADHD, they did not assess whether these factors act as explanatory mechanisms in the association between ADHD symptoms and educational level. We aimed to address this omission by investigating whether having more ADHD symptoms contributes to poorer family functioning and less social support by teachers and classmates, and consequently a lower educational level (i.e., mediation).

### Poor Family Functioning and Lack of Social Support by Teachers and Classmates may Amplify the Association between ADHD Symptoms and Lower Educational Level (i.e., Interaction)

Adverse family functioning and poor relationships with teachers and peers may also amplify the association between ADHD symptoms and lower education, yet past research on this topic is limited. We were able to identify only one study showing weaker associations between ADHD symptoms and lower Grade Point Average (GPA) in adolescents who felt socially accepted by peers (Dvorsky et al., 2018). This suggests that adolescents with low social support by peers and high levels of ADHD symptoms may be at particularly high risk of poorer educational outcomes, as they, for instance, cannot rely on academic support from classmates, for example through sharing resources, such as notes and books, which may reduce the impact of their symptoms on education (Dvorsky et al., 2018). To our knowledge, it has not yet been investigated whether the findings of Dvorsky et al. (2018) concerning fluctuations in GPA extend to long-term outcomes, such as educational track membership. Furthermore, we are not aware of any studies that assessed interactions between family functioning or social support by teachers and ADHD symptoms in affecting adolescent’s educational level. It is conceivable that ADHD symptoms may affect educational level more strongly in the presence of poor family functioning, as poorly functioning families are less able to provide a supportive learning environment at home, for example by helping with homework, or vouch for their children at school. Similarly, ADHD symptoms may be particularly detrimental for the education of adolescents whose relationships with teachers are conflictual, given that adolescents with ADHD frequently have to rely on their teachers for accommodations to meet their academic needs (Harrison et al., 2020).

### Aims of the Study

In the present study, we aimed to contribute to the literature by investigating the role of three important family and school factors (i.e., family functioning, social support by teachers, and social support by classmates) as mediators in the association between ADHD symptoms and (changes in) educational level from early adolescence to young adulthood in the Dutch educational system. While doing so, we allowed for potential interactions between ADHD symptoms and family and school factors using interventional effects for mediation analysis (VanderWeele et al., 2014; Vansteelandt & Daniel, 2017). By using a multi-informant approach, we avoided mono-informant bias and were able to consider both parents’ and adolescents’ views on ADHD symptoms. By covering the entire adolescent period and the transition into young adulthood, we took into account potential changes in the role of parents, teachers, and peers in youngsters’ education over the course of development. Our study sheds new light on how several systems (family, school) around adolescents may interact or contribute to the association between ADHD symptoms and lower educational attainment.

## Materials and Methods

### Study Population

We used data from the first four waves (T1 – T4) of the TRacking Adolescents’ Individual Lives Survey (TRAILS), a population-based prospective cohort study of Dutch adolescents (*n* = 2,229, 49.26% male, 13.50% non-Dutch ethnicity). A detailed description of the cohort can be obtained elsewhere (Oldehinkel et al., 2015). At the beginning of the study, 135 schools in the provinces of Groningen, Friesland, and Drenthe were invited, of which 122 decided to participate. Adolescents were followed between 2000 and 2010, with assessments around age 11, 14, 16, and 19. 

### ADHD Symptoms

ADHD symptoms were assessed using a multi-informant approach, by computing the mean score of the DSM-oriented ADHD symptom scales of the parent-report Child Behavior Checklist (CBCL) and the Youth Self-report (YSR) of the Achenbach System of Empirically Based Assessment (ASEBA), completed at wave 1, 2, and 3 (i.e., around age 11, 14, and 16) (Achenbach & Rescorla, 2001). The YSR and CBCL contain lists of questions on emotional and behavioural problems in the preceding six months, with three response categories: 0 = ‘not true’, 1 = ‘somewhat or sometimes true’, 2 = ‘very or often true’. Sample items from the scales include ‘difficulties concentrating’, ‘not finishing tasks’, and ‘being unable to sit still’. Cronbach’s alphas ranged from 0.68 to 0.74 for the YSR, and from 0.82 to 0.84 for the CBCL. Mean scores of the YSR and CBCL scales (7 items each) were computed separately, and then the mean of both scales was taken, yielding a scale ranging from 0 to 2, with higher scores indicating higher levels of ADHD symptoms. The DSM-oriented scales were constructed based on the ratings of experienced psychiatrists and psychologists in terms of the consistency of each item in the CBCL/YSR with DSM-IV ADHD diagnostic criteria. While the DSM-oriented scales do not measure all DSM symptom criteria of ADHD, and cannot take into account the age of onset, duration, and level of impairment, the scales have been able to distinguish between diagnosed and non-diagnosed children, and are strongly associated with other standardized rating scales, such as the Conners Scales (Achenbach et al., 2003).

### Adolescents’ Educational Level

**Fig. 1:**
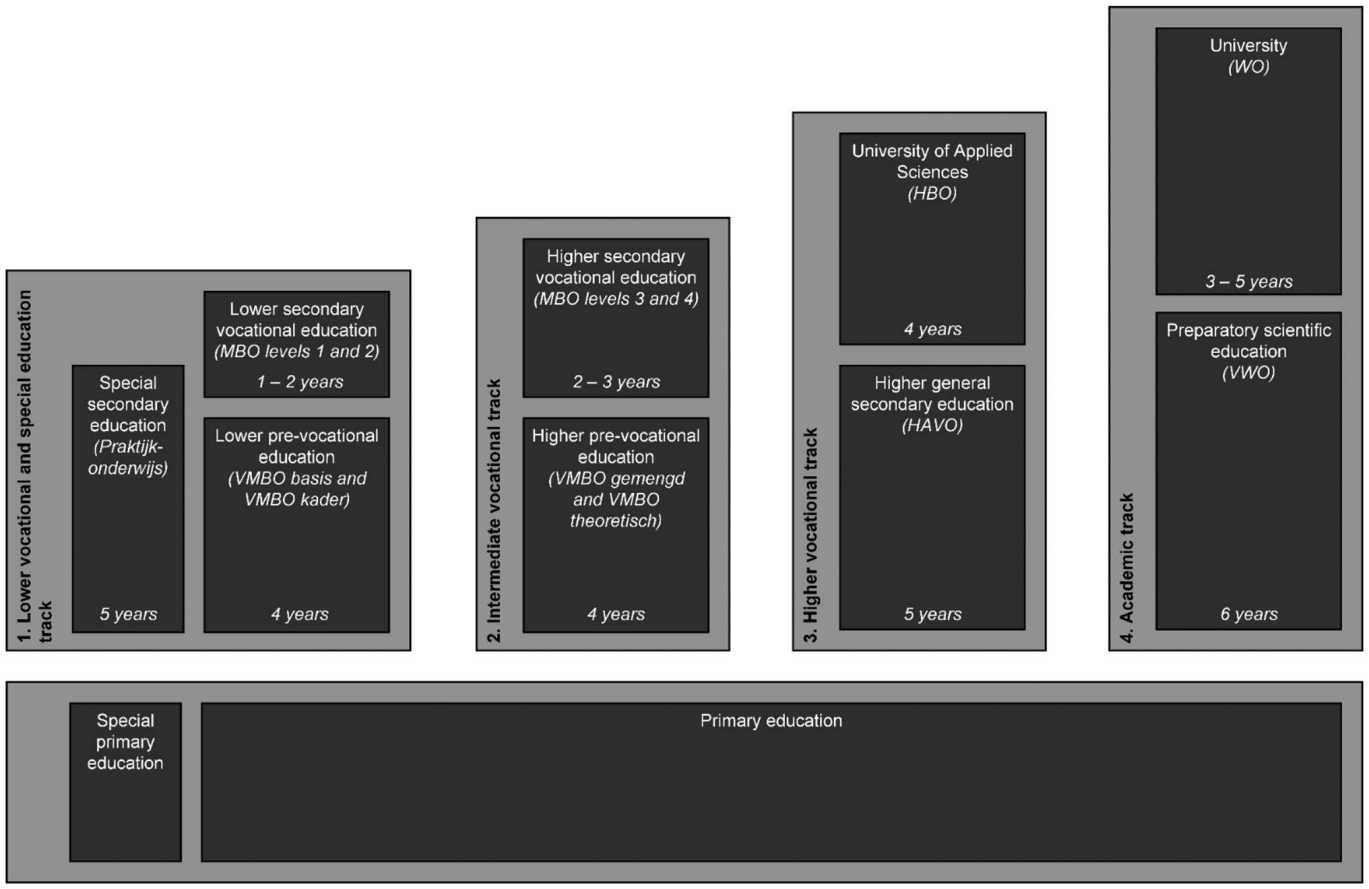
The Dutch educational system

The Dutch educational system is characterized by an early (age 11–12) selection into a particular educational track, based on cognitive tests and the advice of the primary school. There are four tracks in the Dutch educational system, each consisting of a specific type of secondary school followed by tertiary education at the corresponding level (Fig. 1): (1) lower vocational track, (2) intermediate vocational track, (3) higher vocational track, (4) academic track. In addition, there is a special education track, attended by students who are unable to attend regular education. This track was collapsed with the lower vocational track in our analyses. While in secondary education, adolescents can be recommended by their school to move between educational tracks, depending on their academic performance. Furthermore, after attaining specific milestones of their track, students can become eligible to continue their education at a higher track. Overall, a substantial proportion of students is mobile between educational tracks: 24.66% of adolescents moved to a different track between wave 2 and 3 (i.e., between around age 14 and 16), and 25.41% between wave 3 and 4 (i.e., between around age 16 and 19). Educational track membership was assessed at each wave by asking for participants’ current enrolment, as well as their highest completed diploma. Participants who finished the final diploma of a given track received the value corresponding to that level for all subsequent waves, unless they continued education at a higher level. Our measure of educational level allows us to assign a score that represents an age-appropriate measure of educational level as proxy of developing socioeconomic status (SES).

### Family Functioning

Family functioning was assessed at wave 1, 2, and 3 (i.e., around ages 11, 14, and 16) by parent-report using a modified version of the Dutch version of the General Functioning Scale of the McMaster Family Assessment Device (FAD), which is a mean score of 12 items with each four response categories: 1 = ‘totally disagree’, 2 = ‘disagree’, 3 = ‘agree’, 4 = ‘totally agree’. (Bouma et al., 2008; Epstein et al., 1983). We recoded the items of the FAD such that higher scores indicate better family functioning. Six dimensions of family functioning were assessed: communications, problem solving, affective responsiveness, affective involvement, roles, and behaviour control. Example items include ‘being able to count on each other’s support’, ‘trusting each other’, and ‘avoiding talking about one’s fears and worries’ within the family. The scale has demonstrated adequate test–retest reliability, is moderately correlated with other self-report family functioning measures, and has shown utility in differentiating between clinician-rated healthy and unhealthy families (Hamilton & Carr, 2016; Wenniger et al., 1993). The Cronbach’s alpha of the FAD in TRAILS ranged from 0.85 to 0.87.

### Social Support by Teachers

Social support by teachers was assessed at wave 1, 2, and 3 (i.e., around age 11, 14, and 16) by adolescent-report using the mean score of the corresponding affection (4 items) and behavioural confirmation (4 items) subscales adapted from the Social Production Functions (SPF) Questionnaire (Ormel et al., 1997). The response options were 1 = ‘never’, 2 = ‘almost never’, 3 = ‘sometimes’, 4 = ‘almost always’, 5 = ‘always’. Higher scores on the scale indicate higher levels of social support by teachers. Example items include ‘most teachers are satisfied with the way I am’ and ‘I can trust most teachers’. The Cronbach’s alpha ranged from 0.75 to 0.78 for the affection subscale, and from 0.72 to 0.74 for the behavioural confirmation subscale.

### Social Support by Classmates

Social support by classmates was assessed at waves 1, 2, and 3 (i.e., around age 11, 14, and 16) by adolescent-report using the mean score of the corresponding affection (4 items) and behavioural confirmation (4 items) subscales adapted from the Social Production Functions (SPF) Questionnaire (Ormel et al., 1997). The response options were 1 = ‘never’, 2 = ‘almost never’, 3 = ‘sometimes’, 4 = ‘almost always’, 5 = ‘always’). Higher scores on the scale indicate higher levels of social support by classmates. Example items include ‘most classmates help me in case of a problem’ and ‘most classmates like to do things with me’. The Cronbach’s alpha ranged from 0.80 to 0.84 for the affection subscale, and from 0.76 to 0.82 for the behavioural confirmation subscale.

### Covariates

Covariates assessed at baseline around age 11 (wave 1) include children’s IQ, which was estimated using the Block Design and Vocabulary subtests of the Revised Wechsler Intelligence Scale for Children (WISC-R), as well as parents’ socioeconomic status (SES), constructed as the mean score of five indicators (standardized): maternal and paternal educational attainment, maternal and paternal occupational position (according to the International Standard Classification of Occupations), and family income. Furthermore, we included gender and ethnicity as demographic covariates. Children were classified as having non-Dutch ethnicity if at least one of their parents was born outside the Netherlands. Finally, we adjusted for adolescent age, which was measured contemporaneously with each assessment of ADHD symptoms.

### Analytic Approach

**Fig. 2:**
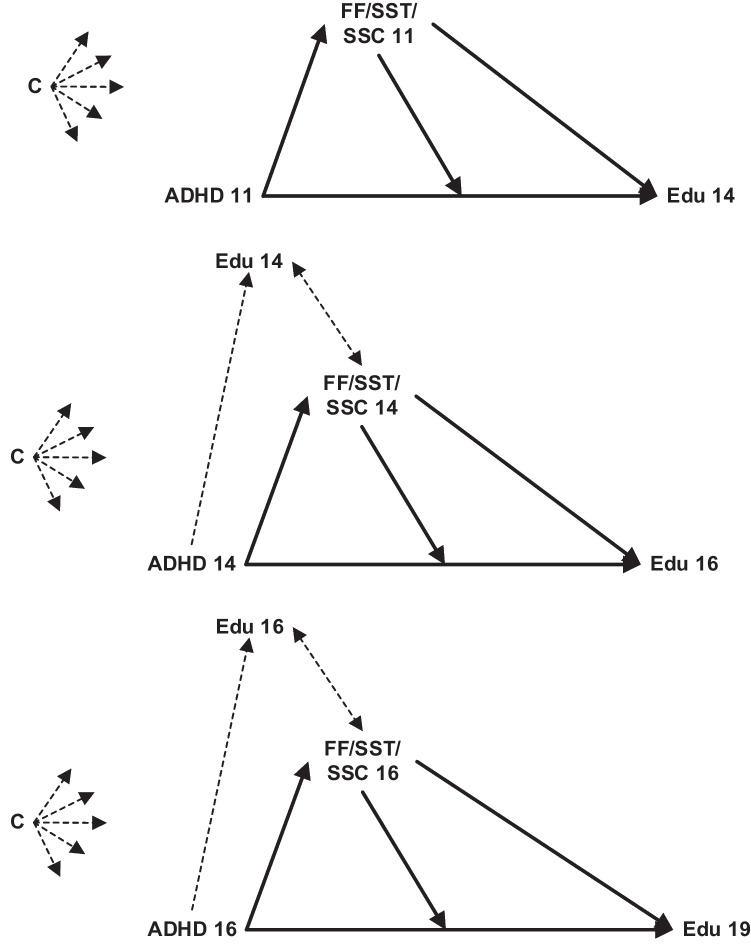
Illustrations of the hypothesized relationships between ADHD symptoms, family and school factors, and educational level across adolescence, as assessed with mediation analysis using interventional effects; Edu = educational level; FF = family functioning; SST = social support by teachers; SSC = social support by classmates; C = covariates, which were included in all regression equations (i.e., gender, ethnicity, IQ, and parental SES measured at baseline, and age assessed in the same wave ADHD symptoms and potential mediators were measured)

First, we computed descriptive statistics of the study population by cross-tabulating baseline characteristics (mean age 11) with early adolescent educational track membership at wave 2 (mean age 14), as well as ADHD symptoms and family and school factors with concurrent educational level from wave 2 to wave 3 (mean age 19). In mediation analyses, we modelled the association between ADHD symptoms and initial educational level in early adolescence (i.e., around age 14). Subsequent changes in educational level were estimated by regressing educational level around 16 and 19 on previous measurements of educational level (i.e., around 14 and 16, respectively). We assessed the potential mediating role of family functioning, and social support by teachers and classmates, all measured concurrently with ADHD symptoms (around ages 11, 14, and 16), in the association between ADHD symptoms and (changes in) education, whilst additionally evaluating interactions between ADHD symptoms and these hypothesized mediators (Fig. 2). Separate models were run for each age category (i.e., between around age 11 and 14, 14 and 16, and 16 and 19) and for each potential mediator (i.e., family functioning, social support by classmates, social support by teachers). It is possible that ADHD symptoms also affect educational level after relatively short amounts of time. In this case, measures of educational level assessed concurrently with ADHD symptoms could function as exposure-induced mediator-outcome confounders, which can cause bias in many types of mediation models. We therefore used interventional effects mediation models, which can still yield valid results by treating these confounders as additional mediators (Chan & Leung, 2022; VanderWeele et al., 2014; Vansteelandt & Daniel, 2017). All continuous variables were z-score transformed to facilitate interpretability of coefficients. If change in education was estimated by regressing educational level on its past value, standardized beta-coefficients of 0.03 were judged as small, 0.07 as medium, and 0.12 as large effects, as recommended by Orth et al. (2022) for longitudinal autoregressive models. For all other estimates from mediator and outcome models, we followed recommendations by Funder & Ozer (2019), who suggested classifying effects of 0.05 as very small, 0.10 as small, 0.20 as medium, 0.30 as large, and 0.40 as very large in psychological research. The effect sizes of indirect effects were given by the proportion mediated (Alwin & Hauser, 1975; Goldstein, 2016).

Attrition analyses showed that at wave 2 3.63% (*N* = 81) of the original participants no longer participated in the study. At wave 3 this was the case for 18.44% (*N* = 411), and at wave 4 for 15.66% (*N* = 349) of the original participants. Adolescents with greater age, male gender, non-Dutch ethnicity, lower educational level and IQ, as well as those from lower SES households were more likely to drop out of the study (Table S1). ADHD symptoms were also related to dropout, but only significantly so at wave 1 (i.e., around age 11). Considering family and school factors, lower family functioning around age 14 was related to increased risks of having left the study by wave 4 (i.e., around age 19). Comparable differences were found between participants with complete information on educational level and those whose educational level was missing or could not be classified (Table S2).

Missing information on educational track membership from wave 2 to 4 was imputed using retrospective event history calendar data collected at wave 3 and wave 5. Participants who were still in elementary education or in a combined class at wave 2 were assigned according to their elementary school teacher’s recommended level. If this information was not available, pupils were classified according to the first track they attended after leaving elementary education or the combined class. It was not possible to classify participants who had not been in education for a longer period, were not classifiable into an educational track (e.g., because of education abroad), whose educational level was assessed incompletely, who did not respond to questions on education, or who had left the educational system permanently (wave 2: *N* = 221, 10.29%; wave 3: *N* = 289, 15.90%; wave 4: *N* = 373, 19.84%). Education was considered as missing for these participants. This missing information together with missing values on all other variables was addressed using multiple imputations by chained equations under fully conditional specification (van Buuren, 2007) and under the assumption of missingness at random. 90 imputed datasets were created with 50 iterations between datasets. Analyses were conducted in STATA 16.1 and in R 4.2.2, making use of the ‘intmed’ (version 0.1.2) package (Chan, 2022) for mediation analyses.

### Sensitivity Analyses

The ‘intmed’ package currently does not support ordinal outcomes. We therefore assessed whether the ordinal nature of our educational variable affected the linear regression results of the mediation models by repeating our analyses using structural equation modelling and the weighted least square means and variance adjusted (WLSMV) estimator in Mplus 8.8. These models allow for ordinal outcomes, while assuming the absence of exposure-mediator interactions (Figs. S1-S3). In mediation models it is usually preferable to measure exposures, mediators, and outcomes in consecutive waves. However, the time lags between measurements in TRAILS are rather long (about three years), which means that adolescents are frequently in different social contexts (e.g., in different classrooms with different teachers and peers) in one wave compared to the next. To adequately assess the consequences of ADHD symptoms, in terms of social support in the classroom and family functioning, it is important to measure these variables within the same social context as ADHD symptoms, necessitating a fairly short time interval between measurements. This is why we modelled exposures and mediators contemporaneously in our main analyses. Nevertheless, we conducted sensitivity analyses in which we allowed for each one wave time lag between measurements of exposures, mediators, and outcomes, to assess whether the choice of time lags affects our results (Figure S4, Table S3).

## Results

### Descriptive Statistics

Table 1 shows the characteristics of TRAILS participants around age 11 according to educational level around age 14. More ADHD symptoms around age 11 were strongly associated with lower education around age 14. Children about to attend the lower educational tracks in early adolescence tended to experience poorer family functioning. No significant differences according to educational level around age 14 were found in terms of social support by teachers and classmates around age 11. Children with less affluent or non-Dutch parents were more commonly selected into the lower educational tracks. Girls more frequently went on to attend the academic and intermediate vocational tracks than boys. Further, higher IQ around age 11 was strongly associated with higher education around age 14. Being in a higher educational track was strongly and inversely associated with ADHD symptoms around age 14 and 16 (Table 2). Overall, with few exceptions, adolescents in the higher educational tracks tended to experience somewhat better family functioning, and more social support by teachers and classmates around 14 and 16.

**Table 1:** Characteristics of adolescents participating in the TRAILS Study (wave 1 – 4, the Netherlands, 2000–2010, *N* = 2,229) at wave 1 (2000–2002) according to educational level at wave 2 (2003–2005)

	All levels		Lower vocational & special education		Intermediate vocational		Higher vocational		Academic	
	*N* = 2,229		*N* = 635		*N* = 497		*N* = 383		*N* = 457	
ADHD symptoms^*^, mean (SD)	0.58	(0.33)	0.69^a^	(0.35)	0.62^b^	(0.32)	0.55^c^	(0.31)	0.43^d^	(0.27)
Family functioning, mean (SD)	3.23	(0.36)	3.16^a^	(0.34)	3.23^b^	(0.38)	3.24^b^	(0.35)	3.32^c^	(0.36)
Social support by teachers, mean (SD)	3.81	(0.70)	3.79^a^	(0.79)	3.82^a^	(0.67)	3.78^a^	(0.66)	3.87^a^	(0.57)
Social support by classmates, mean (SD)	3.58	(0.73)	3.60^a^	(0.82)	3.62^a^	(0.71)	3.53^a^	(0.68)	3.53^a^	(0.64)
Male gender, *N* (%)	1,098	(49.26)	341^**a**^	(53.70)	217^**b**^	(43.66)	196^**a**^	(51.17)	195^**b**^	(42.67)
Non-Dutch ethnicity, *N* (%)	301	(13.50)	108^**a**^	(17.01)	61^**b**^	(12.27)	39^**b**^	(10.18)	45^**b**^	(9.85)
Age, mean (SD)	11.11	(0.56)	11.16^**a**^	(0.56)	11.07^**b**^	(0.54)	11.05^**b**^	(0.56)	11.14^**a**^	(0.56)
Parental socioeconomic status (SES), mean (SD)	–0.05	(0.80)	–0.53^**a**^	(0.70)	–0.16^**b**^	(0.67)	0.21^**c**^	(0.68)	0.55^**d**^	(0.70)
Wechsler Intelligence Deviation Quotient, mean (SD)	97.19	(15.00)	86.05^**a**^	(12.49)	95.20^**b**^	(10.98)	102.68^**c**^	(11.20)	111.14^**d**^	(11.91)

SD = standard deviation; parameters with different superscripts differ significantly from each other at *p* < 0.05, as determined by chi-squared tests (categorical variables) and one-way ANOVAs with pairwise comparisons (continuous variables); higher scores indicate higher SES, higher intelligence, more ADHD symptoms, higher levels of family functioning, and more social support by teachers and classmates

^*^ADHD symptoms were assessed using mean scores of the YSR and CBCL DSM-oriented ADHD symptom scales

**Table 2:** Characteristics of adolescents and young adults in the TRAILS Study (wave 1 – 4, the Netherlands, 2000–2010, *N* = 2,229) according to concurrent educational level

	Wave 2		Wave 3		Wave 4	
*N* participants	2,148		1,818		1,880	
Date range	2003–2005		2005–2008		2008–2010	
Male gender, *N* (%)	1,054	(49.07)	867	(47.69)	898	(47.77)
Educational level, *N* (%)						
Lower vocational & special education	635	(32.20)	349	(22.83)	161	(10.68)
Intermediate vocational	497	(25.20)	405	(26.49)	498	(33.02)
Higher vocational	383	(19.42)	362	(23.68)	475	(31.50)
Academic	457	(23.17)	413	(27.01)	374	(24.80)
ADHD symptoms^*^, mean (SD)						
All levels	0.54	(0.32)	0.52	(0.32)	–	–
Lower vocational & special education	0.63^a^	(0.33)	0.64^a^	(0.34)	–	–
Intermediate vocational	0.58^b^	(0.31)	0.57^b^	(0.32)	–	–
Higher vocational	0.53^c^	(0.32)	0.50^c^	(0.28)	–	–
Academic	0.43^d^	(0.28)	0.40^d^	(0.27)	–	–
Family functioning, mean (SD)					–	–
All levels	3.36	(0.40)	3.35	(0.40)	–	–
Lower vocational & special education	3.26^a^	(0.41)	3.32^a^	(0.44)	–	–
Intermediate vocational	3.36^b^	(0.40)	3.31^a^	(0.41)	–	–
Higher vocational	3.41^b/c^	(0.38)	3.35^a/b^	(0.38)	–	–
Academic	3.44^c^	(0.38)	3.40^b^	(0.38)	–	–
Social support by teachers, mean (SD)						
All levels	3.48	(0.65)	3.43	(0.61)	–	–
Lower vocational & special education	3.47^a^	(0.74)	3.41^a/b^	(0.69)	–	–
Intermediate vocational	3.47^a^	(0.60)	3.44^a/b^	(0.63)	–	–
Higher vocational	3.46^a^	(0.62)	3.37^a^	(0.61)	–	–
Academic	3.56^b^	(0.56)	3.48^b^	(0.48)	–	–
Social support by classmates, mean (SD)						
All levels	3.59	(0.65)	3.57	(0.55)	–	–
Lower vocational & special education	3.50^a^	(0.74)	3.53^a^	(0.65)	–	–
Intermediate vocational	3.61^b^	(0.66)	3.55^a/b^	(0.54)	–	–
Higher vocational	3.63^b^	(0.60)	3.58^a/b^	(0.51)	–	–
Academic	3.64^b^	(0.57)	3.61^b^	(0.49)	–	–
Age, mean (SD)						
All levels	13.57	(0.53)	16.28	(0.71)	–	–
Lower vocational & special education	13.65^a^	(0.52)	16.15^a^	(0.66)	–	–
Intermediate vocational	13.53^b^	(0.56)	16.14^a^	(0.72)	–	–
Higher vocational	13.49^b^	(0.54)	16.25^b^	(0.62)	–	–
Academic	13.55^b^	(0.49)	16.25^b^	(0.54)	–	–

*SD* = standard deviation; parameters with different superscripts differ significantly from each other at *p* < 0.05, as determined by one-way ANOVAs with pairwise comparisons; higher scores indicate more ADHD symptoms, higher levels of family functioning, and more social support by teachers and classmates

^*^ADHD symptoms were assessed using mean scores of the YSR and CBCL DSM-oriented ADHD symptom scales

### Mediation and Interaction Analyses

Mediation analyses (Table 3) revealed small direct effects of ADHD symptoms in childhood (around age 11) on lower educational level around age 14. Similarly, we found medium-sized direct effects of ADHD symptoms around age 14 and 16 on decreases in educational level by around age 16 and 19, respectively. Our models also revealed small associations between ADHD symptoms and lower family functioning, as well as less social support by classmates, and medium-sized to large associations between ADHD symptoms and less social support by teachers, throughout the whole study period. Unexpectedly, in none of our mediation models were family functioning and social support by teachers and classmates associated with (changes in) educational level. Accordingly, the interventional effects mediation models detected no significant indirect effects. Furthermore, we did not find any significant interactions between family functioning, and social support by classmates and teachers and ADHD symptoms.

**Table 3:** Direct and indirect effects of ADHD symptoms on (changes in) educational level in each subsequent wave, as well as selected estimates from mediator and outcome models in the TRAILS Study (wave 1 – 4, the Netherlands, 2000–2010, *N* = 2,229); potential mediators were measured concurrently with ADHD symptoms and evaluated in separate models; linear regression (standardized beta-coefficient, 95% Confidence Interval, *p*-value)

	Family functioning			Social support by teachers			Social support by classmates		
	IE	DE	PM	IE	DE	PM	IE	DE	PM
Direct and indirect effects									
Educational level age 14	0.00 (-0.01–0.01), 0.818	**-0.14** **(-0.17–-0.11),** **< 0.001**	0.01	-0.01 (-0.02**–**0.00), 0.088	**-0.14** **(-0.17–-0.10),** **< 0.001**	0.05	-0.01 (-0.01–0.00), 0.079	**-0.14** **(-0.17–-0.11),** **< 0.001**	0.04
Changes in educational level between around age 14 and 16	0.00 (0.00–0.01), 0.652	**-0.09** **(-0.12–-0.07),** ** < 0.001**	0.01	-0.01 (-0.01**–**0.00), 0.232	**-0.09** **(-0.11–-0.06),** **< 0.001**	0.03	0.00 (-0.01**–**0.00), 0.514	**-0.09** **(-0.11–-0.06),** **< 0.001**	0.01
Changes in educational level between around age 16 and 19	0.00 (-0.01–0.00), 0.491	**-0.08** **(-0.11–-0.05),** **< 0.001**	0.01	-0.01 (-0.02–0.00), 0.179	**-0.07** **(-0.10–-0.04),** **< 0.001**	0.04	0.00 (0.00**–**0.01), 0.696	**-0.08** **(-0.11–-0.05),** **< 0.001**	0.01
	Family functioning			Social support by teachers			Social support by classmates		
Mediator model estimates (hypothesized mediators were measured concurrently with ADHD symptoms)									
ADHD symptoms around age 11	**-0.17 (-0.21–-0.12), < 0.001**			**-0.21 (-0.25–-0.16), < 0.001**			**-0.16 (-0.20–-0.11), < 0.001**		
ADHD symptoms around age 14	**-0.18 (-0.23–-0.14), < 0.001**			**-0.25 (-0.29–-0.21), < 0.001**			**-0.18 (-0.23–-0.14), < 0.001**		
ADHD symptoms around age 16	**-0.18 (-0.23–-0.13), < 0.001**			**-0.30 (-0.35–-0.25), < 0.001**			**-0.14 (-0.19–-0.09), < 0.001**		
	Educational level around age 14			Changes in educational level between around age 14 and 16			Changes in educational level between around age 16 and 19		
Outcome model estimates (outcomes were measured one wave after ADHD symptoms and the hypothesized mediators)									
Family functioning	0.02 (-0.01–0.05), 0.248			-0.01 (-0.04–0.01), 0.314			0.01 (-0.02–0.04), 0.574		
Social support by teachers	0.02 (-0.01–0.06), 0.186			0.02 (-0.01–0.04), 0.232			0.02 (-0.01–0.05), 0.150		
Social support by classmates	0.01 (-0.02–0.05), 0.421			0.01 (-0.01–0.03), 0.446			-0.01 (-0.04–0.02), 0.722		
ADHD × family functioning	-0.02 (-0.06–0.01), 0.118			0.01 (-0.02–0.03), 0.653			0.01 (-0.02–0.03), 0.689		
ADHD × social support by teachers	0.01 (-0.02–0.04), 0.366			0.00 (-0.02–0.03), 0.746			0.00 (-0.02–0.03), 0.810		
ADHD × social support by classmates	0.02 (-0.01–0.05), 0.110			0.00 (-0.02–0.02), 0.911			0.00 (-0.03–0.02), 0.846		

IE = indirect effect; DE = direct effect; PM = proportion mediated; **boldface** denotes statistical significance at *p* < 0.05; all models are adjusted for time-stable covariates measured at baseline (i.e., gender, ethnicity, IQ, parental SES) and age assessed in the same wave ADHD symptoms and potential mediators were measured; past education, which is a potential exposure-induced mediator-outcome confounder (Chan & Leung, 2022; VanderWeele et al., 2014; Vansteelandt & Daniel, 2017), was treated as additional mediator

Results regarding mediation in our sensitivity analysis allowing for ordinal outcomes were consistent with our main analysis (Figs. S1-S3). However, when one wave time lag (approximately 3 years) was allowed between the measurements of ADHD symptoms and family and school factors, we detected negligible to very small but significant associations between more social support by teachers and classmates around age 14 and a higher educational level around age 16, as well as between more social support by teachers around age 16 and increases in educational level by around age 19. Nevertheless, we found only a very small (proportion mediated = 0.05) indirect effect of ADHD symptoms around age 11 on lower education around age 16 via lower levels of social support by classmates around age 14 (Fig. S4 and Table S3).

In order to gain further insight into why we found no mediation when modelling ADHD symptoms and family and school factors contemporaneously, we conducted sequentially adjusted regression analyses of associations between family functioning and social support by teachers and classmates and subsequent (changes in) education (Table S4). These analyses revealed some negligible to small associations between family and school factors and (changes in) education, all of which remained or became significant after adjustment for covariates, but none of which survived further adjustment for ADHD symptoms.

## Discussion

In this study, we investigated the role of family functioning and social support by classmates and teachers as mediators within associations between symptoms of ADHD and educational level, while also evaluating potential interactions between ADHD symptoms and these family and school factors. Significant direct effects revealed that ADHD symptoms were associated with being in a lower educational track in early adolescence, and that adolescents with high levels of ADHD symptoms more frequently decreased in their educational level over the course of adolescence, relative to their peers with low levels of ADHD symptoms, irrespective of family functioning and social support by teachers and peers. In addition, ADHD symptoms were associated with worse family functioning, as well as lower levels of social support by teachers and classmates throughout the whole study period. Yet, significant indirect effects were absent in all but one model in our sensitivity analyses, in which ADHD symptoms around age 11 were allowed to influence social support by classmates approximately three years later. Whenever family and school factors were modelled contemporaneously with ADHD symptoms (i.e., within the same social contexts), no mediation was found. This is likely due to the small magnitude of associations between family and school factors and subsequent (changes in) education after adjustment for covariates, which were even completely absent after also adjusting for concurrent measures of ADHD symptoms. Lastly, we observed no interactions between ADHD symptoms and family and school factors, suggesting that associations between ADHD symptoms and education do not differ in the presence of varying levels of family functioning and social support by teachers and peers. Overall, our results highlight the robustness of the detrimental associations of ADHD symptoms with educational level throughout adolescence, which persist even in the presence of positive social relationships within the family and with teachers and peers.

### Interpretation of Findings

#### Family and School Factors Largely do not Mediate the Association between ADHD Symptoms and Educational Level

Despite robust and consistent relations of ADHD symptoms with both educational level and family and school factors, contrary to our hypotheses, we found little empirical support that these factors contribute much to the association between ADHD symptoms and lower education as mediators. Mediation was largely absent given that we only observed negligible to small associations between family functioning and social support by teachers and classmates and (changes in) education, which were no longer significant following adjustment for covariates and ADHD symptoms, if those symptoms were assessed within the same social contexts as family and school factors. One exception was an extremely small indirect effect via social support by classmates in the sensitivity analysis in which family and school factors were measured approximately three years after ADHD symptoms. This was unexpected, given that we deemed mediation more likely with ADHD symptoms and family and school factors measured at the same time. Our findings contrast past research highlighting the importance of the social environment at home and at school for adolescents’ educational attainment (Blackson, 1995; Lin et al., 2019; Robertson & Reynolds, 2010; Roorda et al., 2017; Roy et al., 2017; Tao et al., 2022; Wentzel et al., 2021).

There are several potential explanations for the discrepancy between our results and past research. First, our measures of family functioning and peer/teacher support may not have sufficiently tapped into the aspects of social relationships contributing most strongly to the association between ADHD symptoms and educational level. Family functioning, for instance, is a rather general measure assessing family climate but may not exactly capture the specific aspects of parenting (e.g., parental involvement in school) important for the education of adolescents (Castro et al., 2015; Masud et al., 2015; Song et al., 2015). Similarly, adolescents may perceive much affection and behavioural confirmation from peers and teachers, which would be reflected in high scores on the SPF scales used here, but may lack more academic support from their classmates (e.g., through note sharing) (Dvorsky et al., 2018) or teachers (e.g., accommodations) (Harrison et al., 2020). Future studies may benefit from including measures of social support that more specifically relate to adolescents’ academic development.

Second, most previous studies have used GPA-based measures (Gallardo et al., 2016; Goguen et al., 2010; Scales et al., 2020; Sebanc et al., 2014; Wentzel & Caldwell, 1997) or standardized testing (Blackson, 1995; Lee, 2012; Li et al., 2020; Liem & Martin, 2011; Lin et al., 2019; Phan & Ngu, 2018; Song et al., 2015) as measures of education. Family and school factors may strongly affect day-to-day fluctuations in GPA, yet these fluctuations might not be substantial enough to cause moving to a lower school type for most adolescents. Still, two studies which, similar to ours, used measures of long-term educational outcomes (mean number of school certificate passes (Woodward & Fergusson, 2000), and degree attainment in young adulthood (Roy et al., 2017)) did find associations. Nevertheless, family and school factors might be more strongly and consistently associated with GPA than long-term educational outcomes.

Lastly, differences between our results and past findings could be explained by divergent approaches to covariate adjustment. For example, most previous studies on social factors and education had a non-clinical focus and therefore did not adjust for, for instance, adolescents’ psychological problems, including ADHD symptoms. We were able to find only two studies controlling for similar covariates to our study, which, in line with our results, both found substantial reductions in associations between family and school factors and educational outcomes after statistical adjustment. Yet, unlike most associations in our study, associations in these studies remained significant. In Sun et al. (2021), the association between family functioning and school disruption was strongly attenuated after adjusting for adolescents’ psychological problems, including ADHD symptoms. Similar results were found by Woodward and Fergusson (2000), who besides ADHD symptoms, like in our study also adjusted for parental SES and IQ, concerning the association between peer relationship problems and later educational attainment (Woodward & Fergusson, 2000). Overall, these results highlight the importance of careful covariate selection when evaluating associations between social factors and educational outcomes.

#### Family and School Factors and ADHD Symptoms do not Interact

We found no interactions of family functioning and social support by teachers and classmates with ADHD symptoms, suggesting that ADHD symptoms are similarly related to education across varying levels of these family and school factors. A potential explanation for this finding is that the current structure of the Dutch educational system might not be able to provide enough support to adolescents with high levels of ADHD symptoms, even if teachers and parents are willing to provide such assistance. For example, classes in most schools might be too large to optimally support students with special needs, including ADHD, for whom small classes of maximally 8 to 15 students may be optimal (Loe & Feldman, 2007). In 2016, in secondary schools in the Netherlands, the academic and higher vocational tracks had an average class size of 27 pupils in the first year, and the intermediate and lower vocational tracks 19 and 12 pupils, respectively (Dutch House of Representatives, 2017). In primary school, the average class size was at 23 pupils (Dutch Ministry of Education, Culture and Science, 2018). It is thus possible that, particularly in primary school and the higher educational tracks, the Dutch system currently cannot sufficiently accommodate the academic needs of adolescents grappling with ADHD symptoms. The effect of insufficient facilitation may be strong, such that educational outcomes do not change for the better even if these adolescents perceive positive relationships with teachers and peers and grow in up in households with a positive family climate. Furthermore, the smaller number of pupils in intermediate vocational and lower vocational tracks may be late in view of reaching higher educational outcomes, and insufficient facilitation in primary school may have already set lower educational outcomes in motion.

### Strengths and Limitations

Key strengths of our study are a high response rate, its long follow-up, and the consistency of measures over time, allowing to capture multiple developmental periods simultaneously (Oldehinkel et al., 2015). By incorporating both parent- and self-reported ADHD symptoms, we were able to avoid mono-informant bias (Martel et al., 2021). Furthermore, we incorporated factors both in the school and home context, which has rarely been done in previous studies. Another strength of our study is our measure of educational level, which is consistent throughout adolescence and young adulthood. The selective educational system of the Netherlands provides an age-appropriate measure of educational attainment, as proxy for developing SES over the course of adolescence. That is, the selection into educational tracks as early as at age 11–12 years means that Dutch adolescents grow up in distinct educational environments that are characterized by different social norms, future expectations, cognitive resources, and occupational prospects — characteristics that are closely related to conceptualizations of SES in adulthood (Schmengler et al., 2021). One could therefore argue that in selective educational systems, such as in the Netherlands, youngsters move into ‘their own’ SES at a much earlier age than in comprehensive systems, such as in Finland or the USA. TRAILS provides a unique opportunity to investigate both the antecedents and consequences, in terms of health-related characteristics, of this differentiation and subsequent intragenerational social mobility in adolescents and young adults (Schmengler et al., 2021).

Some limitations of this study may have affected our results and conclusions. First, we used data from a relatively low-risk population-based sample of adolescents, which means that findings may not extend to high-risk or clinical samples of adolescents diagnosed with ADHD. As we did not use clinical diagnoses, we could not distinguish between the impact of symptoms above and below clinical thresholds. Second, informants might differ in how they judge social relationships. Future studies may take a multi-informant approach when assessing family functioning and social relationships of adolescents with teachers and peers, to take into account differing perspectives on these relationships. Third, attrition might have influenced the results of our study. Although we implemented multiple imputations to manage missing data, higher dropout of adolescents with less favourable conditions (e.g., lower education, parental SES, IQ) may still have affected our results. As these characteristics are also important determinants of adverse outcomes in young adulthood, further research on at-risk groups is necessary (Caspi et al., 2016). Lastly, future research may also consider the reverse-causal path in the association between ADHD and family functioning. For example, past studies have found associations between early-life adversity, including indicators of family dysfunction, and later neurodevelopmental outcomes, including symptoms of ADHD (Xu et al., 2022). Particularly early and severe emotional deprivation may adversely affect children’s neurodevelopment, potentially through epigenetic mechanisms (Sonuga-Barke et al., 2017), yet familial confounding may also contribute to associations between childhood adversity and ADHD symptoms (Carlsson et al., 2021). Future research may employ genetically informed designs to investigate to what extent ADHD symptoms contribute to the association between childhood adversity, including severe family dysfunction, and later educational attainment.

## Conclusion and Implications

Our findings suggest that ADHD symptoms are robustly associated with lower educational attainment over the course of adolescence. Yet, this association was not mediated by general measures of family functioning and social support by teachers and classmates. Furthermore, we found no evidence that these measures amplify the association between ADHD symptoms and lower educational level. General social support and family functioning may still substantially contribute to associations between ADHD symptoms and other functional outcomes, such as mental health (Dvorsky & Langberg, 2016; Karawekpanyawong et al., 2021; Meinzer et al., 2021). For educational attainment, specific aspects of social support proximally related to academic functioning may be more important (e.g., parental involvement in school, note sharing with classmates, accommodations at school). Crucially, research on the role of social factors in associations between ADHD symptoms and functional outcomes is still in its infancy (Dvorsky & Langberg, 2016). Therefore, replication studies are necessary to explore the extent to which our results extend across dimensions of social support and to other, including high-risk, populations. Lastly, our findings may differ from studies on interventions targeting adolescents’ social context, as interventions addressing multiple systems (family, teachers, peers) have shown promise and should not be ignored based on our results (DuPaul et al., 2020; Sibley et al., 2016, 2020). These studies typically have a relatively short follow-up (DuPaul et al., 2020) and may therefore also capture more subtle effects of the social context on academic functioning, which may be missed in studies with long follow-up and focus on long-term educational attainment, like TRAILS.

## Supplementary Information

Below is the link to the electronic supplementary material.

Supplementary file1 (DOCX 753 KB)

## Data Availability

Under the General Data Protection Regulation (GDPR), our dataset is considered pseudonymized rather than anonymized, and is still regarded as personal data. When participants were invited to the cohort more than 20 years ago, they were not asked to give informed consent to make their personal data publicly available in pseudonymized form. As a result of this, legal and ethical restrictions prevent the authors from making data from the TRAILS Study publicly available. For more information about accessing data from the TRAILS Study, please see https://www.trails.nl/en/hoofdmenu/data/data-use. The syntax for our analyses can be obtained from: https://github.com/hschmengler/ADHD-symptoms-and-educational-level-in-adolescents-the-role-of-the-family-teachers-and-peers

## References

[CR1] Achenbach TM, Rescorla L (2001). Manual for the ASEBA school age forms & profiles: an integrated system of multi-informant assessment.

[CR2] Achenbach TM, Dumenci L, Rescorla LA (2003). DSM-oriented and empirically based approaches to constructing scales from the same item pools. Journal of Clinical Child & Adolescent Psychology.

[CR3] Ahmed W, Minnaert A, van der Werf G, Kuyper H (2010). Perceived social support and early adolescents’ achievement: The mediational roles of motivational beliefs and emotions. Journal of Youth and Adolescence.

[CR4] Alwin DF, Hauser RM (1975). The decomposition of effects in path analysis. American Sociological Review.

[CR5] Blackson TC (1995). Temperament and IQ mediate the effects of family history of substance abuse and family dysfunction on academic achievement. Journal of Clinical Psychology.

[CR6] Bouma EM, Ormel J, Verhulst FC, Oldehinkel AJ (2008). Stressful life events and depressive problems in early adolescent boys and girls: The influence of parental depression, temperament and family environment. Journal of Affective Disorders.

[CR7] Carlsson T, Molander F, Taylor MJ, Jonsson U, Bölte S (2021). Early environmental risk factors for neurodevelopmental disorders – a systematic review of twin and sibling studies. Development and Psychopathology.

[CR8] Caspi A, Houts RM, Belsky DW, Harrington H, Hogan S, Ramrakha S, Poulton R, Moffitt TE (2016). Childhood forecasting of a small segment of the population with large economic burden. Nature Human Behaviour.

[CR9] Castro M, Expósito-Casas E, López-Martín E, Lizasoain L, Navarro-Asencio E, Gaviria JL (2015). Parental involvement on student academic achievement: A meta-analysis. Educational Research Review.

[CR10] Chan, G., & Leung, J. (2022). *StatsNotebook CMA Module – an R-based open-source software for causal mediation analysis using the interventional effect approach* [Unpublished manuscript]. Centre for Youth Substance Abuse Research, University of Queensland, Australia.

[CR11] Chan TW, Koo A (2010). Parenting style and youth outcomes in the UK. European Sociological Review.

[CR12] Chan, G. (2022). *intmed: Mediation analysis using interventional effects*. R package version 0.1.2. Retrieved March 03, 2023, from https://CRAN.R-project.org/package=intmed

[CR13] DuPaul GJ, Evans SW, Mautone JA, Owens JS, Power TJ (2020). Future directions for psychosocial interventions for children and adolescents with ADHD. Journal of Clinical Child & Adolescent Psychology.

[CR14] Dutch House of Representatives (2017). *Kamerstuk 31293, nr. 360.* Retrieved September 29, 2022, from https://zoek.officielebekendmakingen.nl/kst-31293-360.html

[CR15] Dutch Ministry of Education, Culture and Science (2018). *Groepsgrootte basisonderwijs 2014–2017.* Retrieved September 29, 2022, from https://duo.nl/open_onderwijsdata/images/groepsgrootte-in-het-basisonderwijs-2014-2017.pdf

[CR16] Dvorsky MR, Langberg JM (2016). A review of factors that promote resilience in youth with ADHD and ADHD symptoms. Clinical Child and Family Psychology Review.

[CR17] Dvorsky MR, Langberg JM, Evans SW, Becker SP (2018). The protective effects of social factors on the academic functioning of adolescents with ADHD. Journal of Clinical Child & Adolescent Psychology.

[CR18] Epstein NB, Baldwin LM, Bishop DS (1983). The McMaster family assessment device. Journal of Marital and Family Therapy.

[CR19] Ewe LP (2019). ADHD symptoms and the teacher–student relationship: A systematic literature review. Emotional and Behavioural Difficulties.

[CR20] Fang G, Chan PWK, Kalogeropoulos P (2020). Social support and academic achievement of Chinese low-income children: A mediation effect of academic resilience. International Journal of Psychological Research (Medellin).

[CR21] Funder DC, Ozer DJ (2019). Evaluating Effect Size in Psychological Research: Sense and Nonsense. Advances in Methods and Practices in Psychological Science.

[CR22] Gallardo LO, Barrasa A, Guevara-Viejo F (2016). Positive peer relationships and academic achievement across early and mid-adolescence. Social Behavior and Personality.

[CR23] Glatz T, Stattin H, Kerr M (2011). Parents’ reactions to youths’ hyperactivity, impulsivity, and attention problems. Journal of Abnormal Child Psychology.

[CR24] Goguen LMS, Hiester MA, Nordstrom AH (2010). Associations among peer relationships, academic achievement, and persistence in college. Journal of College Student Retention: Research, Theory & Practice.

[CR25] Goldstein, N. D. (2016). *Epi Vignettes: Mediation frameworks and analysis*. Retrieved February 13, 2023, from https://www.goldsteinepi.com/blog/epivignettesmediationframeworksandanalysis/index.html

[CR26] Hamilton E, Carr A (2016). Systematic review of self-report family assessment measures. Family Process.

[CR27] Harrison JR, Evans SW, Baran A, Khondker F, Press K, Noel D, Wasserman S, Belmonte C, Mohlmann M (2020). Comparison of accommodations and interventions for youth with ADHD: A randomized controlled trial. Journal of School Psychology.

[CR28] Honkasilta J, Vehkakoski T, Vehmas S (2016). ‘The teacher almost made me cry’ Narrative analysis of teachers' reactive classroom management strategies as reported by students diagnosed with ADHD. Teaching and Teacher Education.

[CR29] Jones HA, Rabinovitch AE, Hubbard RR (2015). ADHD symptoms and academic adjustment to college: The role of parenting style. Journal of Attention Disorders.

[CR30] Karawekpanyawong N, Wongpakaran T, Wongpakaran N, Boonnag C, Siritikul S, Chalanunt S, Kuntawong P (2021). Impact of perceived social support on the relationship between adhd and depressive symptoms among first year medical students: A structural equation model approach. Children.

[CR31] Lawson GM, Owens JS, Mandell DS, Tavlin S, Rufe S, So A, Power TJ (2022). Barriers and facilitators to teachers’ use of behavioral classroom interventions. School Mental Health.

[CR32] Lee J-S (2012). The effects of the teacher–student relationship and academic press on student engagement and academic performance. International Journal of Educational Research.

[CR33] Li L, Peng Z, Lu L, Liao H, Li H (2020). Peer relationships, self-efficacy, academic motivation, and mathematics achievement in Zhuang adolescents: a moderated mediation model. Children and Youth Services Review.

[CR34] Liem GAD, Martin AJ (2011). Peer relationships and adolescents’ academic and non-academic outcomes: Same-sex and opposite-sex peer effects and the mediating role of school engagement. British Journal of Educational Psychology.

[CR35] Lin Y-C, Washington-Nortey P-M, Hill OW, Serpell ZN (2019). Family functioning and not family structure predicts adolescents’ reasoning and math skills. Journal of Child and Family Studies.

[CR36] Loe IM, Feldman HM (2007). Academic and educational outcomes of children with ADHD. Journal of Pediatric Psychology.

[CR37] Lorijn SJ, Engels MC, Huisman M, Veenstra R (2022). Long-term effects of acceptance and rejection by parents and peers on educational attainment: A study from pre-adolescence to early adulthood. Journal of Youth and Adolescence.

[CR38] Martel MM, Eng AG, Bansal PS, Smith TE, Elkins AR, Goh PK (2021). Multiple informant average integration of ADHD symptom ratings predictive of concurrent and longitudinal impairment. Psychological Assessment.

[CR39] Masud H, Thurasamy R, Ahmad MS (2015). Parenting styles and academic achievement of young adolescents: A systematic literature review. Quality & Quantity.

[CR40] Matejevic M, Todorovic J, Jovanovic AD (2014). Patterns of family functioning and dimensions of parenting style. Procedia - Social and Behavioral Sciences.

[CR41] McQuade JD, Becker SP (2020). Peer functioning in adolescents with ADHD. ADHD in adolescents: Development, assessment, and treatment.

[CR42] Meinzer MC, Felton JW, Oddo LE, Rubin KH, Chronis-Tuscano A (2021). Do ADHD symptoms and relationship quality with mothers and best friends across high school predict depressive symptoms for adolescents?. Journal of Attention Disorders.

[CR43] Moen ØL, Hedelin B, Hall-Lord ML (2014). Parental perception of family functioning in everyday life with a child with ADHD. Scandinavian Journal of Public Health.

[CR44] Oldehinkel AJ, Rosmalen JGM, Buitelaar JK, Hoek HW, Ormel J, Raven D, Reijneveld SA, Veenstra R, Verhulst FC, Vollebergh WAM, Hartman CA (2015). Cohort profile update: The TRacking Adolescents’ Individual Lives Survey (TRAILS). International Journal of Epidemiology.

[CR45] Ormel J, Lindenberg S, Steverink N, Vonkorff M (1997). Quality of life and social production functions: A framework for understanding health effects. Social Science & Medicine.

[CR46] Orth, U., Meier, L. L., Bühler, J. L., Dapp, L. C., Krauss, S., Messerli, D., & Robins, R. W. (2022). Effect size guidelines for cross-lagged effects. *Psychological Methods*. Advance online publication. 10.1037/met0000499 10.1037/met0000499 35737548

[CR47] Patrick H, Ryan AM, Kaplan A (2007). Early adolescents' perceptions of the classroom social environment, motivational beliefs, and engagement. Journal of Educational Psychology.

[CR48] Phan HP, Ngu BH (2018). An examination of social and psychological influences on academic learning: A focus on self-esteem, social relationships, and personal interest. Social Psychology of Education.

[CR49] Robertson DL, Reynolds AJ (2010). Family profiles and educational attainment. Children and Youth Services Review.

[CR50] Rogers M, Bélanger-Lejars V, Toste JR, Heath NL (2015). Mismatched: ADHD symptomatology and the teacher–student relationship. Emotional and Behavioural Difficulties.

[CR51] Roorda DL, Jak S, Zee M, Oort FJ, Koomen HMY (2017). Affective teacher–student relationships and students' engagement and achievement: A meta-analytic update and test of the mediating role of engagement. School Psychology Review.

[CR52] Roy, A., Hechtman, L., Arnold, L. E., Swanson, J. M., Molina, B. S. G., Sibley, M. H., Howard, A. L., & Group, M. T. A. C. (2017). Childhood predictors of adult functional outcomes in the Multimodal Treatment Study of attention-deficit/hyperactivity disorder (MTA). *Journal of the American Academy of Child and Adolescent Psychiatry*, 56, 687-695.e687. 10.1016/j.jaac.2017.05.020 10.1016/j.jaac.2017.05.020 PMC5555165 28735698

[CR53] Scales PC, Van Boekel M, Pekel K, Syvertsen AK, Roehlkepartain EC (2020). Effects of developmental relationships with teachers on middle-school students’ motivation and performance. Psychology in the Schools.

[CR54] Schmengler H, Peeters M, Stevens GWJM, Kunst AE, Hartman CA, Oldehinkel AJ, Vollebergh WAM (2021). Educational level, attention problems, and externalizing behaviour in adolescence and early adulthood: The role of social causation and health-related selection—the TRAILS study. European Child & Adolescent Psychiatry. Advance Online Publication..

[CR55] Schroeder VM, Kelley ML (2009). Associations between family environment, parenting practices, and executive functioning of children with and without ADHD. Journal of Child and Family Studies.

[CR56] Sebanc AM, Guimond AB, Lutgen J (2014). Transactional relationships between Latinos’ friendship quality and academic achievement during the transition to middle school. Journal of Early Adolescence.

[CR57] Sibley MH, Graziano PA, Kuriyan AB, Coxe S, Pelham WE, Rodriguez L, Sanchez F, Derefinko K, Helseth S, Ward A (2016). Parent–teen behavior therapy + motivational interviewing for adolescents with ADHD. Journal of Consulting and Clinical Psychology.

[CR58] Sibley MH, Morley C, Rodriguez L, Coxe SJ, Evans SW, Morsink S, Torres F (2020). A peer-delivered intervention for high school students with impairing ADHD symptoms. School Psychology Review.

[CR59] Song J, Bong M, Lee K, Kim S-I (2015). Longitudinal investigation into the role of perceived social support in adolescents’ academic motivation and achievement. Journal of Educational Psychology.

[CR60] Sonuga-Barke EJS, Kennedy M, Kumsta R, Knights N, Golm D, Rutter M, Maughan B, Schlotz W, Kreppner J (2017). Child-to-adult neurodevelopmental and mental health trajectories after early life deprivation: The young adult follow-up of the longitudinal English and Romanian Adoptees study. Lancet.

[CR61] Spera C (2005). A review of the relationship among parenting practices, parenting styles, and adolescent school achievement. Educational Psychology Review.

[CR62] Stubbs NS, Maynard D-MB (2017). Academic self-efficacy, school engagement and family functioning, among postsecondary students in the Caribbean. Journal of Child and Family Studies.

[CR63] Sun L, Semovski V, Stewart SL (2021). A study of risk factors predicting school disruption in children and youth living in Ontario. Canadian Journal of School Psychology.

[CR64] Tao Y, Meng Y, Gao Z, Yang X (2022). Perceived teacher support, student engagement, and academic achievement: A meta-analysis. Educational Psychology.

[CR65] Theule J, Wiener J, Tannock R, Jenkins JM (2010). Parenting stress in families of children with ADHD: A meta-analysis. Journal of Emotional and Behavioral Disorders.

[CR66] van Buuren S (2007). Multiple imputation of discrete and continuous data by fully conditional specification. Statistical Methods in Medical Research.

[CR67] VanderWeele, T. J., Vansteelandt, S., & Robins, J. M. (2014). Effect decomposition in the presence of an exposure-induced mediator-outcome confounder. *Epidemiology*, *25, *300-306. 10.1097/EDE.0000000000000034 10.1097/EDE.0000000000000034 PMC4214081 24487213

[CR68] Vansteelandt S, Daniel RM (2017). Interventional effects for mediation analysis with multiple mediators. Epidemiology.

[CR69] Wenniger, W. F. M. d. B., Hageman, W. J. J. M., & Arrindell, W. A. (1993). Cross-national validity of dimensions of family functioning: first experiences with the Dutch version of the McMaster Family Assessment Device (FAD). *Personality and Individual Differences,* 14, 769-781.10.1016/0191-8869(93)90090-P

[CR70] Wentzel KR, Caldwell K (1997). Friendships, peer acceptance, and group membership: Relations to academic achievement in middle school. Child Development.

[CR71] Wentzel KR, Jablansky S, Scalise NR (2021). Peer social acceptance and academic achievement: A meta-analytic study. Journal of Educational Psychology.

[CR72] Wiener J, Daniels L (2015). School experiences of adolescents with attention-deficit/hyperactivity disorder. Journal of Learning Disabilities.

[CR73] Wiener J, Biondic D, Grimbos T, Herbert M (2016). Parenting stress of parents of adolescents with attention-deficit hyperactivity disorder. Journal of Abnormal Child Psychology.

[CR74] Woodward LJ, Fergusson DM (2000). Childhood peer relationship problems and later risks of educational under-achievement and unemployment. Journal of Child Psychology and Psychiatry and Allied Disciplines.

[CR75] Wymbs BT, Pelham WE, Molina BS, Gnagy EM, Wilson TK, Greenhouse JB (2008). Rate and predictors of divorce among parents of youths with ADHD. Journal of Consulting and Clinical Psychology.

[CR76] Xu Y, Ajdacic-Gross V, Müller M, Buadze A, Seifritz E, Kleim B, von Känel R, Wagner E-YN, Strippoli M-PF, Castelao E, Preisig M, Vandeleur CL (2022). Childhood adversity patterns differentially cluster with mental disorders and socioeconomic indicators in a large Swiss community sample. Comprehensive Psychiatry.

[CR77] Zendarski N, Sciberras E, Mensah F, Hiscock H (2017). Academic achievement and risk factors for adolescents with attention-deficit hyperactivity disorder in middle school and early high school. Journal of Developmental and Behavioral Pediatrics.

