# Supplementary material for: ADHD Symptoms and Educational Level in Adolescents: The Role of the Family, Teachers, and Peers
Source: Res Child Adolesc Psychopathol. 2023 Mar 23;51(7):1051–66. doi: 10.1007/s10802-023-01047-y (PMC10271900; doi:10.1007/s10802-023-01047-y)
Supplement: Supplementary file 1 — Supplementary file1 (DOCX 753 KB) [file 10802_2023_1047_MOESM1_ESM.docx]

# **ONLINE SUPPLEMENT**

**Table S1: Attrition analysis – characteristics of young adults remaining in the TRAILS Study (wave 1 – 4, Netherlands, 2000–2010, N = 2,229) at wave 4, compared to participants who had dropped out of the cohort between wave 1 and wave 4**

|  | **Participants remaining in TRAILS by wave 4** | | **Drop-outs** | | **P-value** |
| --- | --- | --- | --- | --- | --- |
| **N participants (%)** | 1,880 | (84.34) | 349 | (15.66) |  |
| **Baseline characteristics** |  |  |  |  |  |
| Male gender, N (%) | 898 | (47.77) | 200 | (57.31) | 0.001 |
| Non-Dutch ethnicity, N (%) | 212 | (11.28) | 89 | (25.50) | <0.001 |
| Parental socioeconomic status (SES), mean (SD) | 0.04 | (0.78) | -0.53 | (0.77) | <0.001 |
| Wechsler Intelligence Deviation Quotient (IQ), mean (SD) | 98.61 | (14.67) | 89.45 | (14.40) | <0.001 |
| **Educational level, mean (SD)** |  |  |  |  |  |
| *Wave 2* | 2.45 | (1.15) | 1.57 | (0.84) | <0.001 |
| *Wave 3* | 2.60 | (1.10) | 1.56 | (0.85) | <0.001 |
| **ADHD symptoms, mean (SD)** |  |  |  |  |  |
| *Wave 1* | 0.57 | (0.33) | 0.61 | (0.36) | 0.035 |
| *Wave 2* | 0.54 | (0.32) | 0.54 | (0.34) | 0.970 |
| *Wave 3* | 0.52 | (0.31) | 0.55 | (0.37) | 0.469 |
| **Family functioning, mean (SD)** |  |  |  |  |  |
| *Wave 1* | 3.23 | (0.36) | 3.20 | (0.37) | 0.147 |
| *Wave 2* | 3.38 | (0.39) | 3.27 | (0.42) | 0.001 |
| *Wave 3* | 3.35 | (0.40) | 3.39 | (0.44) | 0.362 |
| **Social support by teachers, mean (SD)** |  |  |  |  |  |
| *Wave 1* | 3.82 | (0.68) | 3.79 | (0.76) | 0.480 |
| *Wave 2* | 3.49 | (0.65) | 3.44 | (0.68) | 0.322 |
| *Wave 3* | 3.43 | (0.60) | 3.48 | (0.66) | 0.418 |
| **Social support by classmates, mean (SD)** |  |  |  |  |  |
| *Wave 1* | 3.58 | (0.72) | 3.61 | (0.77) | 0.502 |
| *Wave 2* | 3.60 | (0.63) | 3.52 | (0.78) | 0.079 |
| *Wave 3* | 3.58 | (0.55) | 3.51 | (0.63) | 0.219 |
| **Age, mean (SD)** |  |  |  |  |  |
| *Wave 1* | 11.09 | (0.56) | 11.21 | (0.54) | <0.001 |
| *Wave 2* | 13.55 | (0.53) | 13.69 | (0.52) | <0.001 |
| *Wave 3* | 16.27 | (0.70) | 16.42 | (0.82) | 0.015 |

*SD = standard deviation.*

*P-values were computed using chi-squared tests for categorical variables and two-sample t-tests for continuous variables.*

**Table S2: Characteristics of participants with classifiable educational level compared to those with missing/unclassifiable educational level from wave 2 to 4 in the TRAILS Study**

**(wave 1 – 4, the Netherlands, 2000–2010, N = 2,229)**

|  | **N participants per wave** |  | **Male gender** |  | **Non-Dutch ethnicity** |  | **Parental**  **socioeconomic**  **status (SES)** | | **WechslerIntelli-gence Deviation Quotient (IQ)** | | **Concurrent ADHD symptoms*** |  | **Concurrent family functioning** |  | **Concurrent aocial support by teachers** |  | **Concurrent social support by classmates** | | **Concurrent age** |  |
| --- | --- | --- | --- | --- | --- | --- | --- | --- | --- | --- | --- | --- | --- | --- | --- | --- | --- | --- | --- | --- |
|  | **N** | **(%)** | **N** | **(%)** | **N** | **(SD)** | **Mean** | **(SD)** | **Mean** | **(SD)** | **Mean** | **(SD)** | **Mean** | **(SD)** | **Mean** | **(SD)** | **Mean** | **(SD)** | **Mean** | **(SD)** |
| **Wave 2** | 2,148 | (100) |  |  |  |  |  |  |  |  |  |  |  |  |  |  |  |  |  |  |
| *Education complete* | 1,927 | (89.71) | 924 | (47.95) | 237 | (0.56) | -0.03 | (0.80) | 97.58 | (15.02) | 0.55 | (0.32) | 3.36 | (0.40) | 3.49 | (0.65) | 3.59 | (0.66) | 13.57 | (0.53) |
| *Education unclassifiable/missing* | 221 | (10.29) | 130 | (58.82) | 35 | (0.53) | -0.07 | (0.75) | 96.01 | (13.44) | 0.50 | (0.33) | 3.37 | (0.39) | 3.43 | (0.69) | 3.59 | (0.63) | 13.58 | (0.53) |
| *P-value* |  |  |  | 0.002 |  | 0.344 |  | 0.531 |  | 0.138 | 0.067 |  | 0.802 |  | 0.216 |  | 0.882 |  | 0.608 |  |
| **Wave 3** | 1,818 | (100) |  |  |  |  |  |  |  |  |  |  |  |  |  |  |  |  |  |  |
| *Education complete* | 1,529 | (84.10) | 704 | (46.04) | 175 | (0.56) | 0.08 | (0.78) | 99.75 | (14.85) | 0.51 | (0.31) | 3.35 | (0.40) | 3.43 | (0.60) | 3.57 | (0.55) | 16.20 | (0.64) |
| *Education unclassifiable/missing* | 289 | (15.90) | 163 | (56.40) | 36 | (0.57) | -0.32 | (0.78) | 91.25 | (13.22) | 0.55 | (0.33) | 3.32 | (0.40) | 3.44 | (0.65) | 3.60 | (0.62) | 16.70 | (0.90) |
| *P-value* |  |  |  | 0.001 |  | 0.004 |  | <0.001 |  | <0.001 | 0.221 |  | 0.303 |  | 0.832 |  | 0.448 |  | <0.001 |  |
| **Wave 4** | 1,880 | (100) |  |  |  |  |  |  |  |  |  |  |  |  |  |  |  |  |  |  |
| *Education complete* | 1,507 | (80.16) | 671 | (44.53) | 160 | (0.57) | 0.13 | (0.76) | 100.34 | (14.52) | 0.45 | (0.33) | 3.33 | (0.39) | - | - | - | - | 19.03 | (0.58) |
| *Education unclassifiable/missing* | 373 | (19.84) | 227 | (60.86) | 52 | (0.52) | -0.33 | (0.74) | 91.58 | (13.13) | 0.44 | (0.34) | 3.27 | (0.41) | - | - | - | - | 19.28 | (0.64) |
| *P-value* |  |  |  | <0.001 |  | 0.693 |  | <0.001 |  | <0.001 | 0.608 |  | 0.035 |  | - | - | - | - | <0.001 |  |

*SD = standard deviation.*

*P-values were computed using chi-squared tests for categorical variables and 2-sample t-tests for continuous variables.*

** ADHD symptoms at wave 4 were assessed by self-report only.*

**
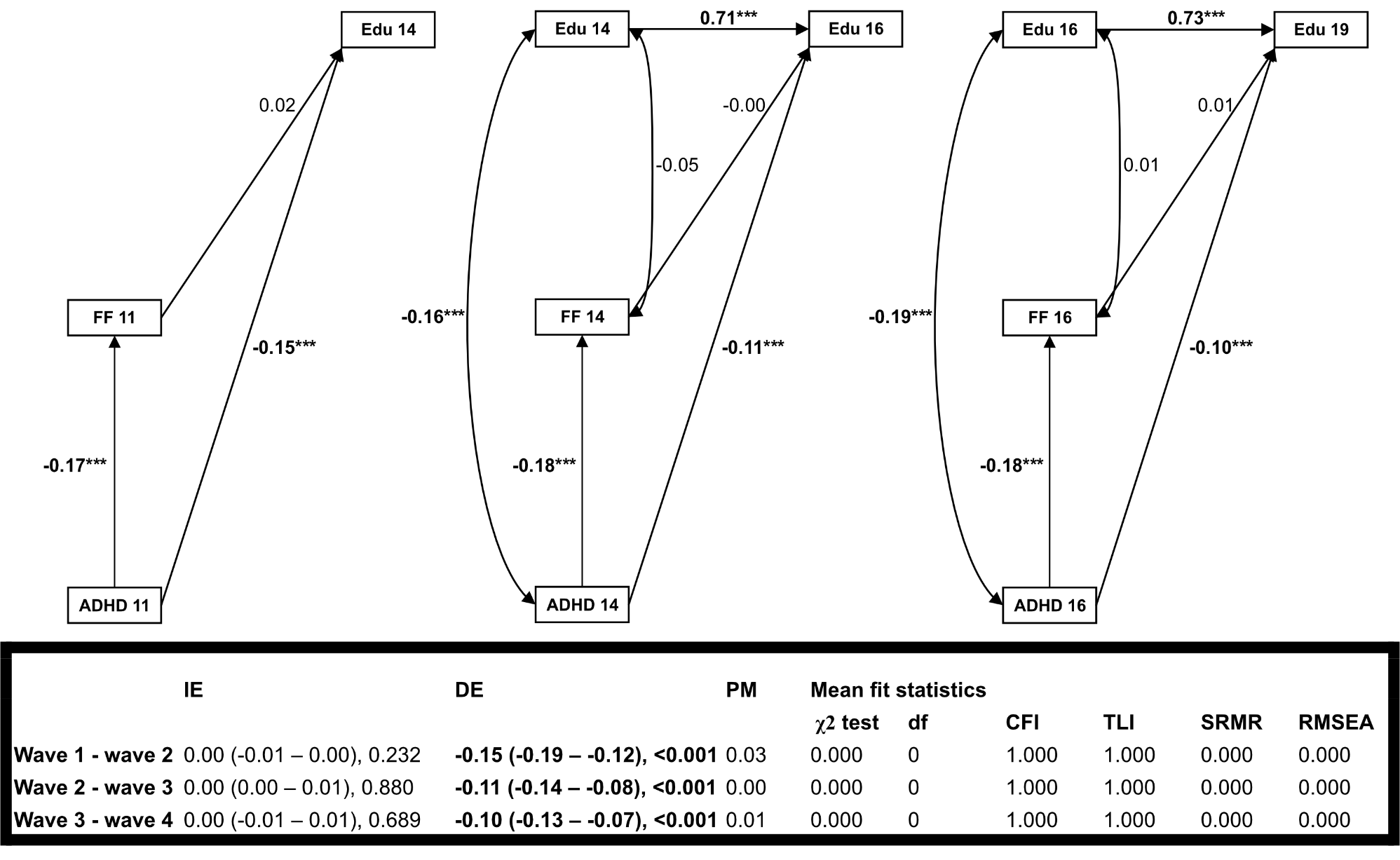
Figure S1: Direct and indirect effects of ADHD symptoms on (changes in) educational level in each subsequent wave in the TRAILS study (wave 1 – 4, the Netherlands, 2000–2010, N = 2,229); family functioning (FF; potential mediator) was measured concurrently with ADHD symptoms; linear and probit regression (WLSMV estimator; standardized beta-coefficient, 95% Confidence Interval, p-value).**

*IE = indirect effect; DE = direct effect; PM = proportion mediated; Edu = educational level; FF = family functioning; * p<0.05; ** p<0.01; *** p<0.001.* ***Boldface*** *denotes statistical significance at p<0.05. All regressions were adjusted for time-stable covariates measured at baseline (i.e., gender, ethnicity, IQ, parental SES) and age assessed in the same wave ADHD symptoms and potential mediators were measured.*

**
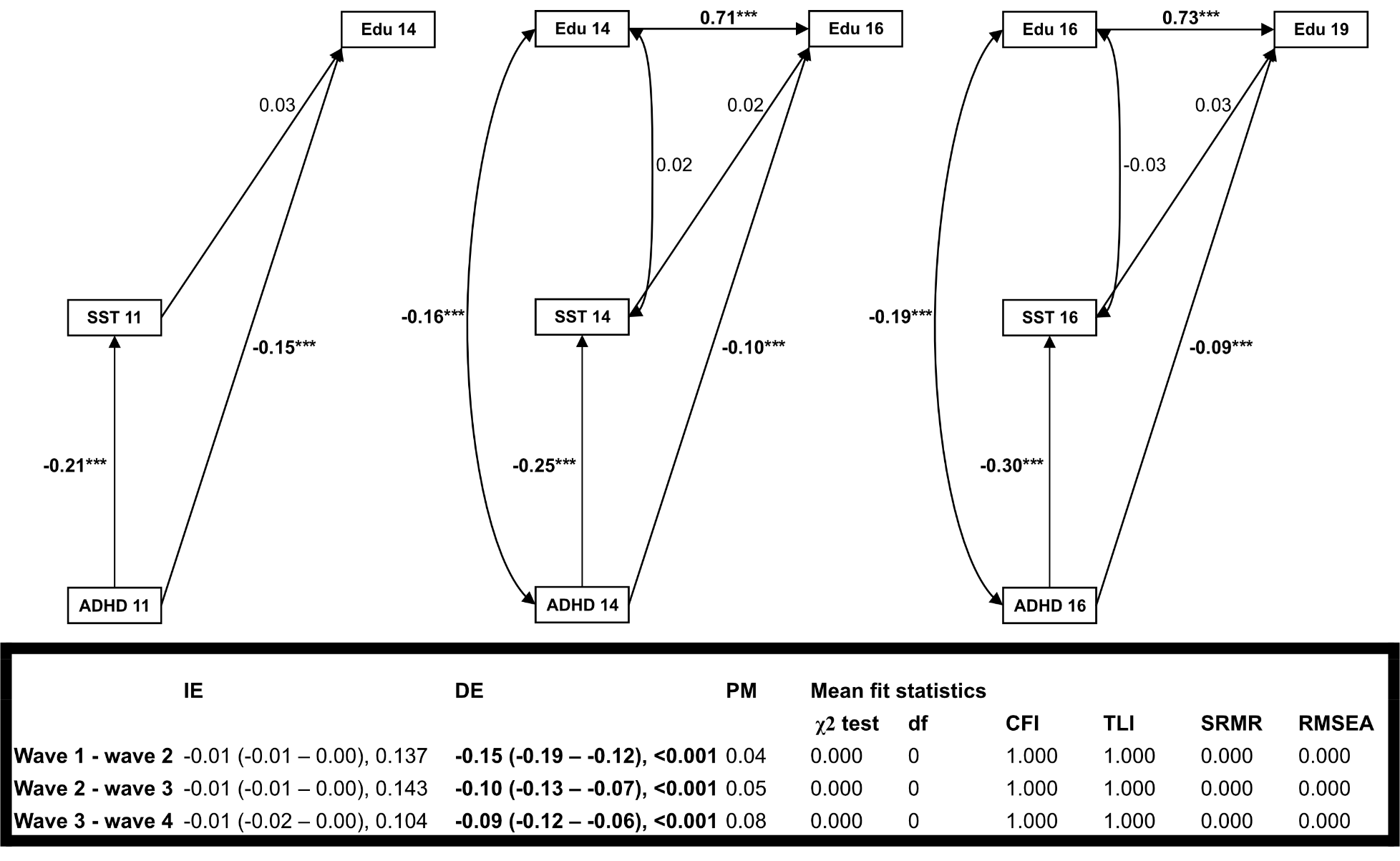
Figure S2: Direct and indirect effects of ADHD symptoms on (changes in) educational level in each subsequent wave in the TRAILS study (wave 1 – 4, the Netherlands, 2000–2010, N = 2,229); social support by teachers (SST; potential mediator) was measured concurrently with ADHD symptoms; linear and probit regression (WLSMV estimator; standardized beta-coefficient, 95% Confidence Interval, p-value).**

*IE = indirect effect; DE = direct effect; PM = proportion mediated; Edu = educational level; SST = social support by teachers; * p<0.5; ** p<0.01; *** p<0.001.* ***Boldface*** *denotes statistical significance at p<0.05. All regressions were adjusted for time-stable covariates measured at baseline (i.e., gender, ethnicity, IQ, parental SES) and age assessed in the same wave ADHD symptoms and potential mediators were measured.*

*
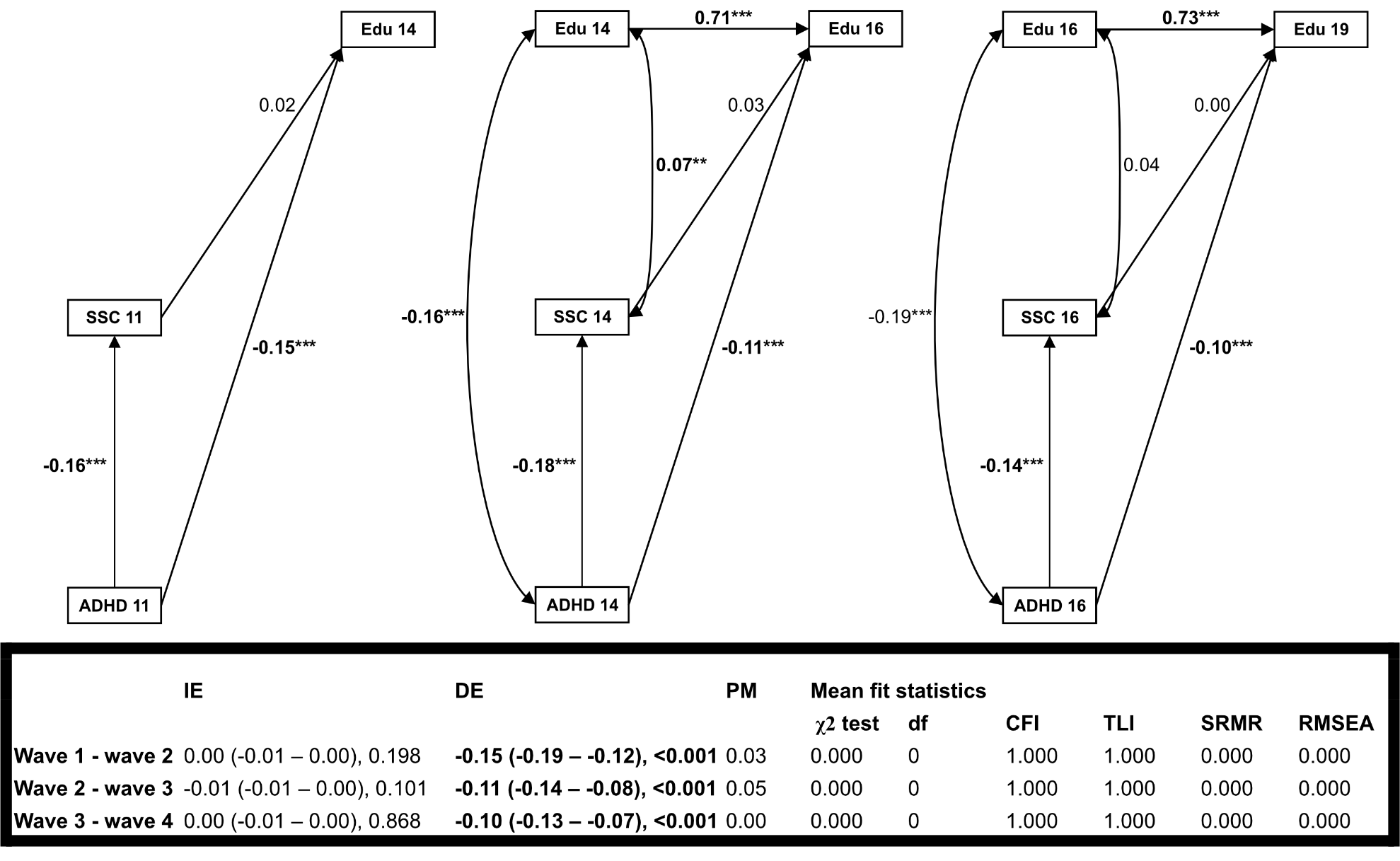
***Figure S3: Direct and indirect effects of ADHD symptoms on (changes in) educational level in each subsequent wave in the TRAILS study (wave 1 – 4, the Netherlands, 2000–2010, N = 2,229); social support by classmates (SSC; mediator) was measured concurrently with ADHD symptoms; linear and probit regression (WLSMV estimator; standardized beta-coefficient, 95% Confidence Interval, p-value).**

*IE = indirect effect; DE = direct effect; PM = proportion mediated; Edu = educational level; SSC = social support by classmates; * p<0.5; ** p<0.01; *** p<0.001. Boldface denotes statistical significance at p<0.05. All regressions were adjusted for time-stable covariates measured at baseline (i.e., gender, ethnicity, IQ, parental SES) and age assessed in the same wave ADHD symptoms and potential mediators were measured.*

**Figure S4: Illustrations of the hypothesized relationships between ADHD symptoms, family and school factors, and educational level across adolescence, allowing each one wave time lag between the measurements of exposure, mediators, and outcomes.**

*Edu = education; FF = family functioning; SST = social support by teachers; SSC = social support by classmates; C = covariates, which were included in all regression equations (i.e., gender, ethnicity, IQ, and parental SES measured at baseline, and age assessed in the same wave ADHD symptoms and potential mediators were measured).*

**Table S3: Direct and indirect effects of ADHD symptoms on (changes in) educational level two waves later, as well as selected estimates from mediator and outcome models in the TRAILS study (wave 1 – 4, the Netherlands, 2000–2010, N = 2,229); potential mediators were evaluated in separate models and measured one wave after ADHD symptoms; linear regression (standardized beta-coefficient, 95% Confidence Interval, p-value).**

|  | Family functioning | | | Social support by teachers | | | Social support by classmates | | |
| --- | --- | --- | --- | --- | --- | --- | --- | --- | --- |
|  | IE | DE | PM | IE | DE | PM | IE | DE | PM |
| Direct and indirect effects |  |  |  |  |  |  |  |  |  |
| Educational level around age 16 | 0.00  (-0.01 – 0.01), 0.480 | **-0.18**  **(-0.21** – **-0.14), <0.001** | 0.01 | -0.01  (-0.02 – 0.00), 0.078 | **-0.17**  **(-0.21 – -0.14),**  **<0.001** | 0.04 | **-0.01**  **(-0.02 – 0.00), 0.023** | **-0.17**  **(-0.20 – -0.13),**  **<0.001** | 0.05 |
| Changes in educational level between around age 14 and 19 | 0.00  (-0.01 – 0.00), 0.259 | **-0.11**  **(-0.14, -0.07), <0.001** | 0.02 | -0.01  (-0.02 – 0.00), 0.055 | **-0.10**  **(-0.13 -0.07),**  **<0.001** | 0.04 | 0.00  (-0.01 **–** 0.01),  0.809 | **-0.11**  **(-0.14 – -0.08),**  **<0.001** | 0.01 |
|  | Family functioning | | | Social support by teachers | | | Social support by classmates | | |
| Mediator model estimates *(hypothesized mediators were measured one wave after ADHD symptoms)* |  |  |  |  |  |  |  |  |  |
| ADHD symptoms around age 11 | **-0.15 (-0.20 – -0.11), <0.001** | | | **-0.17 (-0.22 – -0.13), <0.001** | | | **-0.18 (-0.23 – -0.14), <0.001** | | |
| ADHD symptoms around age 14 | **-0.13 (-0.18 – -0.08), <0.001** | | | **-0.18 (-0.23 – -0.13), <0.001** | | | **-0.17 (-0.22 – -0.12), <0.001** | | |
|  | Educational level around age 16 | | | Changes in educational level between around age 14 and 19 | | |  | | |
| Outcome model estimates  *(outcomes were measured two waves after ADHD symptoms and one wave after the hypothesized mediators)* |  |  |  |  |  |  |  |  |  |
| Family functioning | 0.02 (-0.02 – 0.05), 0.353 | | | 0.01 (-0.02 – 0.04), 0.562 | | |  | | |
| Social support by teachers | **0.04 (0.01 – 0.07), 0.025** | | | **0.04 (0.01 – 0.08), 0.006** | | |  | | |
| Social support by classmates | **0.05 (0.02 – 0.08), 0.005** | | | 0.01 (-0.02 – 0.04), 0.536 | | |  | | |
| ADHD × family functioning | 0.00 (-0.03 – 0.03), 0.971 | | | 0.02 (-0.01 – 0.05), 0.310 | | |  | | |
| ADHD × social support by teachers | 0.00 (-0.03 – 0.03), 0.886 | | | 0.00 (-0.03 **–** 0.02), 0.768 | | |  | | |
| ADHD × social support by classmates | 0.00 (-0.03 – 0.04), 0.813 | | | -0.02 (-0.04 – 0.01), 0.297 | | |  | | |

*IE = indirect effect; DE = direct effect; PM = proportion mediated.* ***Boldface*** *denotes statistical significance at p<0.05. All models are adjusted for time-stable covariates measured at baseline (i.e., gender, ethnicity, parental SES) and age assessed in the same wave ADHD symptoms and potential mediators were measured. Past education, which is a potential exposure-induced mediator-outcome confounder (Chan & Leung, 2022; VanderWeele et al., 2014; Vansteelandt & Daniel, 2017), was treated as additional mediator.*

**Table S4: Associations of family functioning and social support by teachers and classmates with (changes in) education in each subsequent wave in the TRAILS study (wave 1 – 4, the Netherlands, 2000–2010, N = 2,229), adjusted for different sets of covariates; linear regression (standardized beta-coefficient, 95% Confidence Interval, p-value)**

|  | Educational level age 14 | Changes in educational level between around age 14 and 16 | Changes in educational level between around age 16 and 19 |
| --- | --- | --- | --- |
| Models for family functioning |  |  |  |
| Model 1 |  |  |  |
| Family functioning | **0.16 (0.12 – 0.20), <0.001** | 0.00 (-0.02 – 0.03), 0.749 | 0.03 (-0.00 – 0.06), 0.063 |
| Past education | - | **0.85 (0.83 – 0.87), <0.001** | **0.87 (0.84 – 0.89), <0.001** |
| Model 2 |  |  |  |
| Family functioning | **0.04 (0.01 – 0.07), 0.014** | 0.00 (-0.02 – 0.03), 0.793 | 0.02 (-0.01 – 0.05), 0.142 |
| Past education | - | **0.78 (0.75 – 0.81), <0.001** | **0.77 (0.74 – 0.81), <0.001** |
| Model 3 |  |  |  |
| Family functioning | 0.02 (-0.01 – 0.05), 0.269 | -0.01 (-0.04 – 0.01), 0.286 | 0.01 (-0.02 – 0.04), 0.620 |
| Past education | - | **0.76 (0.73 – 0.79), <0.001** | **0.75 (0.72 – 0.79), <0.001** |
| ADHD symptoms | **-0.14 (-0.17 – -0.11), <0.001** | **-0.09 (-0.11 – -0.06), <0.001** | **-0.08 (-0.11 – -0.05), <0.001** |
| ADHD × family functioning | -0.02 (-0.05 – 0.01), 0.123 | 0.01 (-0.01 – 0.03), 0.458 | 0.01 (-0.02 – 0.03), 0.688 |
| Models for social support by teachers |  |  |  |
| Model 1 |  |  |  |
| Social support by teachers | 0.03 (-0.02 – 0.07), 0.215 | **0.03 (0.01 – 0.05), 0.018** | **0.04 (0.01 – 0.06), 0.011** |
| Past education | - | **0.85 (0.83 – 0.87), <0.001** | **0.87 (0.84 – 0.89), <0.001** |
| Model 2 |  |  |  |
| Social support by teachers | **0.05 (0.02 – 0.08), 0.002** | **0.03 (0.01 – 0.06), 0.004** | **0.04 (0.02 – 0.07), 0.002** |
| Past education | - | **0.78 (0.75 – 0.81), <0.001** | **0.77 (0.74 – 0.81), <0.001** |
| Model 3 |  |  |  |
| Social support by teachers | 0.02 (-0.01 – 0.05), 0.185 | 0.01 (-0.01 – 0.04), 0.244 | 0.02 (-0.01 – 0.05), 0.136 |
| Past education | - | **0.76 (0.73 – 0.79), <0.001** | **0.75 (0.72 – 0.79), <0.001** |
| ADHD symptoms | **-0.14 (-0.17 – -0.10), <0.001** | **-0.08 (-0.11 – -0.06), <0.001** | **-0.08 (-0.11 – -0.04), <0.001** |
| ADHD × social support by teachers | 0.01 (-0.02 – 0.04), 0.360 | 0.00 (-0.02 – 0.03), 0.703 | -0.00 (-0.02 – 0.02), 0.973 |
| Models for social support by classmates |  |  |  |
| Model 1 |  |  |  |
| Social support by classmates | **-0.05 (-0.10 -0.01), 0.017** | 0.02 (-0.00 – 0.05), 0.051 | 0.00 (-0.03 – 0.03), 0.796 |
| Past education | - | **0.85 (0.83 – 0.87), <0.001** | **0.87 (0.84 – 0.89), <0.001** |
| Model 2 |  |  |  |
| Social support by classmates | **0.04 (0.00 – 0.07), 0.028** | **0.02 (0.00 – 0.05), 0.038** | 0.01 (-0.02 – 0.04), 0.587 |
| Past education | - | **0.78 (0.75 – 0.81), <0.001** | **0.77 (0.74 – 0.81), <0.001** |
| Model 3 |  |  |  |
| Social support by classmates | 0.01 (-0.02 – 0.05), 0.405 | 0.01 (-0.01 – 0.03), 0.424 | -0.00 (-0.03 – 0.03), 0.867 |
| Past education | - | **0.76 (0.73 – 0.79), <0.001** | **0.75 (0.72 – 0.79), <0.001** |
| ADHD symptoms | **-0.14 (-0.17 – -0.10), <0.001** | **-0.09 (-0.11 – -0.06), <0.001** | **-0.08 (-0.11 – -0.05), <0.001** |
| ADHD × social support by classmates | 0.02 (-0.01 – 0.06), 0.108 | 0.00 (-0.02 – 0.02), 0.841 | -0.00 (-0.03 – 0.02), 0.793 |

***Boldface*** *denotes statistical significance at p<0.05. Coefficients in Models 2 and 3 are additionally adjusted for time-stable covariates measured at baseline (i.e., gender, ethnicity, IQ, parental SES) and age assessed in the same wave ADHD symptoms and potential mediators were measured.*

**REFERENCES**

Chan, G., & Leung, J. (2022). *StatsNotebook CMA Module – an R-based open-source software for causal mediation analysis using the interventional effect approach* [Unpublished manuscript]. Centre for Youth Substance Abuse Research, University of Queensland, Australia.

VanderWeele, T. J., Vansteelandt, S., & Robins, J. M. (2014). Effect decomposition in the presence of an exposure-induced mediator-outcome confounder. *Epidemiology*, 25. doi: 10.1097/EDE.0000000000000034

Vansteelandt, S., & Daniel, R. M. (2017). Interventional effects for mediation analysis with multiple mediators. *Epidemiology*, 28, 258-265. doi: 10.1097/EDE.0000000000000596
